# *Annona muricata* (Soursop) and Hematopoiesis: Ethnomedicinal Insights, Immunomodulatory Mechanisms, and Translational Challenges

**DOI:** 10.3390/antiox15050579

**Published:** 2026-05-03

**Authors:** Fatma Matano, Amiya Patra

**Affiliations:** 1 De Madison Clinic, P.O. Box 84560, Mombasa 80100, Kenya; 2 Peninsula Medical School, University of Plymouth, Plymouth PL6 8BU, UK

**Keywords:** *Annona muricata*, soursop, hematopoiesis, immunomodulation, ethnomedicine, natural products, inflammation

## Abstract

*Annona muricata* (soursop) is a tropical medicinal plant widely used in traditional medicine across Africa, the Caribbean, and parts of South America. While its ethnomedicinal applications span a range of conditions, including infections, inflammation, and anemia-related symptoms, its potential relevance to hematopoiesis has not been systematically examined. This narrative review synthesizes ethnomedicinal knowledge, phytochemical composition, and experimental evidence to explore the biological plausibility by which *A. muricata* may indirectly influence hematopoietic processes. Bioactive constituents of *A. muricata*, including flavonoids, polyphenols, and acetogenins, have demonstrated antioxidant, anti-inflammatory, and immunomodulatory properties in preclinical models. These effects are particularly relevant given the established roles of oxidative stress and chronic inflammation in disrupting hematopoietic stem and progenitor cell function and bone marrow homeostasis. Rather than proposing direct erythropoietic activity, this review emphasizes indirect, marrow-supportive mechanisms through which *A. muricata* may contribute to the preservation of hematopoietic function under conditions of physiological or inflammatory stress. The limitations of the current evidence base, including the predominance of in vitro and animal studies and the absence of direct hematopoietic endpoints in humans, are critically discussed. Overall, this review provides a cautious, integrative framework linking *A. muricata* bioactivity to hematopoietic regulation and highlights key gaps that must be addressed before any translational or clinical relevance can be established.

## 1. Introduction

Hematopoiesis is a tightly regulated physiological process through which hematopoietic stem and progenitor cells (HSPCs) within the bone marrow differentiate into mature blood cell lineages, including erythrocytes, leukocytes, and platelets [1,2,3]. This dynamic process is essential for immune competence, oxygen transport, hemostasis, and maintenance of systemic homeostasis. Disruption of hematopoietic balance can result in a wide spectrum of clinical conditions, ranging from anemia and immunodeficiency to severe cytopenias and bone marrow failure syndromes [1].

Globally, hematological disorders represent a substantial public health burden, particularly in low- and middle-income countries (LMICs) [4]. Anemia alone affects more than two billion people worldwide and remains a leading cause of morbidity among women of reproductive age and children [5,6]. In addition to nutritional deficiencies, conditions such as chemotherapy-induced cytopenias, chronic inflammatory marrow suppression, infectious diseases including malaria, and post-viral syndromes contribute significantly to impaired blood cell production and immune dysfunction, disproportionately impacting resource-limited settings.

At the cellular and molecular levels, impaired hematopoiesis is strongly influenced by oxidative stress, chronic inflammation, and immune dysregulation within the bone marrow microenvironment. Elevated levels of reactive oxygen species (ROS) disrupt hematopoietic stem and progenitor cell (HSPC) quiescence, promote premature differentiation, and induce apoptosis, ultimately compromising long-term blood cell production [3,7,8,9]. Hematopoietic stem cell function is highly sensitive to inflammatory cues, with cytokine signaling and innate immune pathways playing a central role in regulating self-renewal, differentiation, and stress responses within the bone marrow niche [2,10,11,12].

In parallel, sustained exposure to pro-inflammatory cytokines, particularly tumor necrosis factor-α (TNF-α) and interleukin-6 (IL-6), suppresses progenitor cell proliferation, alters lineage commitment, and interferes with iron homeostasis and erythropoiesis [2,13]. Together, oxidative and inflammatory stressors create a hostile marrow environment that underlies anemia of chronic disease, infection-related cytopenias, and inflammation-driven bone marrow suppression [13,14,15].

In response to these challenges, there is growing scientific interest in plant-based modulators of hematopoiesis and immune function, with natural products rich in antioxidants, anti-inflammatory compounds, and immunomodulatory phytochemicals increasingly explored as adjunctive or supportive strategies to preserve bone marrow integrity and promote blood formation, particularly in settings where access to conventional therapies is limited [16,17,18].

*Annona muricata* L. (Annonaceae), commonly known as soursop or graviola, is a widely used ethnomedicinal plant across Africa, the Caribbean, and South America. It has traditionally been employed in the management of febrile illnesses, fatigue, infections, parasitic diseases, and generalized weakness, and remains deeply embedded in community-based healthcare systems [19,20]. Contemporary biomedical research has demonstrated antioxidant, anti-inflammatory, cytotoxic, and immunomodulatory properties of *A. muricata*, largely attributed to its diverse phytochemical composition [21,22,23]. However, despite extensive investigation into its anticancer and metabolic effects, its potential relevance to hematopoiesis and blood cell regulation remains comparatively underexplored. This review critically examines the ethnomedicinal use, phytochemical composition, mechanistic pathways, and emerging preclinical evidence supporting the potential role of Annona muricata in hematopoiesis and blood-related disorders, while highlighting key translational challenges and research gaps.

## 2. Methodology of the Review

This narrative review was conducted to synthesize existing ethnomedicinal and biomedical evidence regarding the potential role of Annona muricata in hematopoiesis and blood-related disorders. A comprehensive literature search was performed using multiple electronic databases to ensure broad coverage of relevant studies [4,17].

### 2.1. Databases and Search Strategy

A comprehensive literature search was conducted using the following electronic databases: PubMed, Scopus, Web of Science, Google Scholarand the African Journals Online (AJOL).

The search covered publications from January 2000 to June 2025, reflecting both traditional knowledge documentation and contemporary biomedical research. Search terms included combinations of the following keywords: “Annona muricata”, “soursop”, “hematopoiesis”, “bone marrow”, “anemia”, “blood cells”, “erythropoiesis”, “immunomodulation”, “oxidative stress”and “inflammation”.

Boolean operators (AND/OR) were applied to refine searches and identify studies addressing both phytochemical properties and hematological relevance [16,24]. In addition, reference lists of relevant reviews and primary articles were manually screened to identify additional pertinent studies and authoritative reports [17,25].

### 2.2. Inclusion and Exclusion Criteria

Inclusion criteria comprised:

Peer-reviewed articles published in English, in vitro and in vivo experimental studies, and ethnomedicinal and pharmacological studies.

Reviews and articles reporting antioxidant, anti-inflammatory, immunomodulatory, or hematological regulatory mechanisms relevant to *A. muricata*, providing a conceptual framework for understanding how bioactive plant compounds may influence hematopoiesis [1,2,3,18,26,27].

Exclusion criteria included:

Non-scientific reports and anecdotal claims, studies unrelated to blood biology or immune function, articles lacking sufficient methodological detail, and duplicate publications.

### 2.3. Data Synthesis

Given the heterogeneity of study designs, methodologies, and reported outcomes, a narrative synthesis approach was employed rather than a systematic or meta-analytic method [7,28]. Selected studies were grouped thematically and critically analyzed to explore links between traditional uses, phytochemical composition, mechanistic pathways, and emerging evidence related to hematopoiesis [10,21,29,30].

This approach enabled integration of ethnomedicinal knowledge with contemporary experimental findings, providing a coherent framework for understanding the potential role of *Annona muricata* in blood-related conditions while highlighting key knowledge gaps requiring further investigation [13,31,32,33].

## 3. Ethnomedicinal Use of *Annona muricata* in Blood-Related Conditions

Ethnomedicinal practices across Africa, the Caribbean, and parts of South America have long utilized Annona muricata for conditions associated with blood health, immune strength, and post-illness recovery. Although traditional descriptions rarely employ biomedical terminology such as “hematopoiesis,” many reported indications correspond to clinical conditions involving impaired blood cell production, immune suppression, or anemia-related fatigue [34,35,36,37]. Importantly, these uses are typically based on orally administered preparations, most commonly aqueous leaf decoctions or infusions, with dosage and duration determined empirically.

### 3.1. Traditional Use in Anemia and Blood Weakness

In several African ethnomedical systems, *A. muricata* leaves and fruit are traditionally used to manage conditions described as “blood weakness,” chronic fatigue, and pallor—symptoms consistent with anemia [20,38]. Leaf decoctions are commonly administered following prolonged illness, malaria, or nutritional deficiency, particularly in settings where access to conventional hematinic therapies is limited.

Malaria-associated anemia, a major public health burden in sub-Saharan Africa, represents a key context in which *A. muricata* is utilized. In such settings, the plant is often administered as a supportive therapy during recovery, suggesting a perceived role in restoring strength and vitality rather than directly stimulating erythropoiesis [20,35].

### 3.2. Post-Illness and Post-Partum Recovery

Traditional use of A. muricata extends to post-partum recovery, particularly in East and West African settings, where it is administered following childbirth to support recovery from blood loss and physiological stress. Preparations are typically given as leaf infusions or decoctions over several days to weeks.

Similarly, individuals recovering from infectious diseases are often given A. muricata-based preparations to improve appetite, energy, and overall vitality [21,39]. While these effects are not explicitly framed in hematological terms, they may reflect indirect support of recovery processes associated with immune and metabolic restoration.

### 3.3. Immune Strengthening and Febrile Illnesses

*A. muricata* is also widely used in the management of febrile illnesses, respiratory infections, and inflammatory conditions. These applications suggest a role in immune modulation, which may indirectly influence hematopoiesis through regulation of inflammatory mediators known to suppress bone marrow function [25,40,41].

In Caribbean and South American traditional medicine, leaf infusions are frequently consumed to “cleanse the blood,” improve circulation, and enhance immune resilience [16,20,38]. Although these descriptions are non-specific, they indicate a consistent perception of systemic and hematological benefits across diverse cultural contexts.

### 3.4. Linking Ethnomedicine to Hematopoietic Relevance

Collectively, these ethnomedicinal practices suggest that *A. muricata* has long been associated with conditions involving fatigue, immune compromise, and recovery from physiological stress. While such uses do not provide direct mechanistic evidence, they offer a valuable framework for hypothesis generation and guide scientific inquiry [1].

The consistent use of aqueous leaf preparations in anemia-like conditions, post-illness recovery, and immune-related disorders provides a plausible basis for investigating the plant’s phytochemical constituents and their potential indirect effects on hematopoiesis. When interpreted alongside emerging preclinical evidence, these traditional observations support further exploration of *A. muricata* within a translational research framework [18,27].

## 4. Phytochemical Profile of *Annona muricata* Relevant to Hematopoiesis

The biological activity of *Annona muricata* is attributed to a diverse spectrum of phytochemical constituents, including annonaceous acetogenins, flavonoids, and phenolic compounds, many of which have been experimentally demonstrated to modulate oxidative stress, inflammatory pathways, and cellular metabolism—processes critically involved in hematopoietic regulation and bone marrow homeostasis [21,32,42,43,44]. Importantly, the distribution and concentration of these compounds vary according to plant part (leaves, fruit, seeds) and extraction method, factors that significantly influence their biological activity and translational relevance.

### 4.1. Acetogenins

Annonaceous acetogenins, predominantly isolated from the leaves and seeds of *A. muricata*, represent a unique class of long-chain polyketide-derived fatty acid derivatives. Among these, compounds such as annonacin and annomuricin have been extensively studied for their potent bioactivity. These compounds exert their primary effect through inhibition of mitochondrial Complex I (NADH:ubiquinone oxidoreductase), leading to reduced ATP production and altered cellular energy metabolism [28,42,45].

Supporting this, experimental studies have demonstrated that acetogenins such as annonacin exert potent cytotoxic effects through inhibition of mitochondrial Complex I, resulting in dose-dependent cellular toxicity in various in vitro and in vivo models [38].

From a hematopoietic perspective, acetogenins exhibit a dose-dependent dual effect. At higher concentrations, mitochondrial inhibition may impair HSPC viability and proliferation. Conversely, at lower or controlled concentrations, modulation of mitochondrial activity and reduction in pathological cellular proliferation may contribute to a more regulated bone marrow environment, particularly under conditions of malignancy or chronic inflammation [2,26,46]. This dualistic behavior highlights the importance of dose standardization, extract characterization, and targeted delivery in any potential translational application.

### 4.2. Flavonoids and Polyphenols

Flavonoids and polyphenolic compounds constitute a major bioactive fraction of *A. muricata*, particularly within the leaves and fruit pulp. Identified compounds include quercetin, kaempferol, rutin, and various phenolic acids, which have been extensively studied in experimental models for their antioxidant and anti-inflammatory properties [21,24,47,48].

In vitro studies have demonstrated that polyphenol-rich extracts of *A. muricata* significantly reduce reactive oxygen species (ROS) levels and inhibit lipid peroxidation in cellular models, including macrophages and epithelial cells. These effects are mediated through both direct free radical scavenging and upregulation of endogenous antioxidant defense systems such as superoxide dismutase (SOD) and catalase.

Oxidative stress is a critical determinant of hematopoietic stem cell fate, with elevated ROS levels driving premature differentiation, cellular senescence, and apoptosis of HSPCs [3,49,50,51,52,53,54,55]. By attenuating oxidative stress, flavonoids and polyphenols in *A. muricata* may help preserve stem cell quiescence, maintain bone marrow niche integrity, and support long-term hematopoietic function. Supporting this, experimental studies using *A. muricata* leaf extracts have demonstrated significant antioxidant activity, including reduction in reactive oxygen species and inhibition of lipid peroxidation in vitro, as reported in preclinical studies [43,56,57].

Furthermore, several polyphenolic compounds have been shown to modulate inflammatory signaling pathways, including inhibition of NF-κB activation and reduction in pro-inflammatory cytokine production. Given the well-established role of inflammation in suppressing erythropoiesis and altering lineage commitment, these anti-inflammatory effects provide an additional mechanistic link between *A. muricata* phytochemicals and hematopoietic regulation [29,30].

## 5. Mechanistic Pathways Linking *Annona muricata* to Hematopoiesis

Hematopoiesis is a tightly regulated biological process governed by interactions between hematopoietic stem and progenitor cells (HSPCs), the bone marrow microenvironment, immune signaling pathways, and systemic metabolic status [58,59]. Disruption of this balance, through oxidative stress, inflammation, infection, malignancy, or pharmacological toxicity, leads to impaired blood cell production. Emerging evidence suggests that oxidative stress is a crucial regulator of hematopoiesis. *Annona muricata* may influence several of these regulatory pathways, thereby exerting indirect or supportive effects on hematopoiesis [37,60].

### 5.1. Modulation of Oxidative Stress in the Bone Marrow Niche

Oxidative stress plays a critical role in hematopoietic dysfunction and is a key regulator of hematopoietic stem cell fate, with excessive ROS driving stem cell dysfunction and exhaustion [3,49,50,51,52]. Inflammatory and infectious stressors induce emergency hematopoiesis and lineage skewing [18,61,62,63]. Elevated reactive oxygen species (ROS) levels impair HSPC self-renewal, promote premature differentiation, and induce apoptosis within the bone marrow niche [26,53,54,55]. Antioxidant systems are therefore essential for maintaining normal hematopoietic homeostasis [64]. Supporting this, experimental evidence indicates that modulation of oxidative stress pathways plays a critical role in preserving hematopoietic stem cell function under pathological conditions.

Polyphenol-rich extracts of *A. muricata* have demonstrated significant antioxidant activity in experimental models, including reduction in reactive oxygen species (ROS) and inhibition of lipid peroxidation [21,24]. By attenuating oxidative stress, these compounds may protect hematopoietic stem and progenitor cells from ROS-mediated damage and preserve bone marrow niche integrity [28,51,52,65].

### 5.2. Anti-Inflammatory Regulation of Hematopoietic Suppression

Chronic inflammation is a major contributor to hematopoietic impairment, particularly in anemia of chronic disease, malignancy-associated cytopenias, and post-viral syndromes. Pro-inflammatory cytokines such as TNF-α, IL-1β, and IL-6 suppress erythropoiesis, disrupt iron metabolism, and impair progenitor cell proliferation [10,13,14,26,63,66,67,68]. Experimental studies have demonstrated that *A. muricata* extracts can suppress inflammatory signaling pathways, including inhibition of NF-κB activation and reduction in pro-inflammatory cytokine production [14,21,27,69]. Given the central role of cytokines such as TNF-α and IL-6 in suppressing erythropoiesis and altering iron metabolism, these effects may contribute to a more permissive hematopoietic environment.

### 5.3. Immunomodulatory Effects and Leukocyte Regulation

Inflammatory and interferon-mediated signaling pathways are now recognized as key regulators of hematopoietic stem cell fate under both physiological and pathological conditions [18,60,62,67]. The immune system and hematopoiesis are intrinsically linked, with immune cell turnover and activation directly influencing bone marrow demand, niche composition, and lineage differentiation. Preclinical studies have reported modulation of macrophage activity, lymphocyte proliferation, and neutrophil responses following exposure to *A. muricata* extracts [21,41], supporting its role in immune-mediated regulation of hematopoiesis.

Alterations in immune transcriptional programs, particularly those involving nuclear factor of activated T cells (NFATc1), may further modulate marrow immune niches and leukopoiesis, providing a mechanistic bridge between immunomodulatory phytochemicals and hematopoietic outcomes [27]. NFAT family transcription factors play a central role in lymphocyte development and function, and NFATc1 activity has been directly implicated in B-cell and T-cell differentiation pathways [10,27,70,71,72].

In this context, the immunomodulatory effects of *A. muricata* may indirectly influence hematopoietic regulation through immune-mediated mechanisms, contributing to balanced leukopoiesis, particularly in conditions characterized by immune exhaustion or dysregulated immune activation [73,74,75,76].

### 5.4. Protection Against Myelosuppression and Cytotoxic Injury

Chemotherapy and certain infectious diseases induce bone marrow suppression through direct cytotoxicity and mitochondrial dysfunction [2,50]. Chronic interferon signaling has been shown to impair hematopoietic stem cell maintenance and survival, linking sustained immune activation to bone marrow dysfunction [2,26].

While acetogenins are known to induce mitochondrial dysfunction at higher concentrations, their potential role in modulating pathological proliferation and oxidative stress at lower concentrations remains theoretical and requires further experimental validation [28,29]. Although direct evidence specific to *A. muricata* remains limited, its phytochemical profile supports further investigation into its potential role as an adjunctive protective agent against marrow toxicity [77,78,79].

### 5.5. Potential Influence on Lineage-Specific Hematopoiesis

Through combined antioxidant, anti-inflammatory, and immunomodulatory mechanisms, *A. muricata* may exert indirect effects across multiple hematopoietic lineages [18,60]:

- Erythropoiesis: via reduction in inflammatory inhibition and oxidative damage;
- Leukopoiesis: through immune regulation and cytokine balance;
- Thrombopoiesis: indirectly via marrow niche protection and reduced inflammatory suppression.

These effects are likely to be indirect and supportive rather than stimulatory, emphasizing the plant’s potential role as a complementary modulator rather than a primary hematopoietic agent.

### 5.6. Conceptual Framework

Collectively, the mechanistic pathways suggest that *A. muricata* may contribute to hematopoietic support by restoring physiological conditions necessary for normal blood cell production. Rather than acting through direct stimulation of hematopoietic stem cells, its effects appear to be mediated through preservation of marrow integrity, immune balance, and metabolic stability [50,51,52,55,60,80,81].

**Figure 1:**
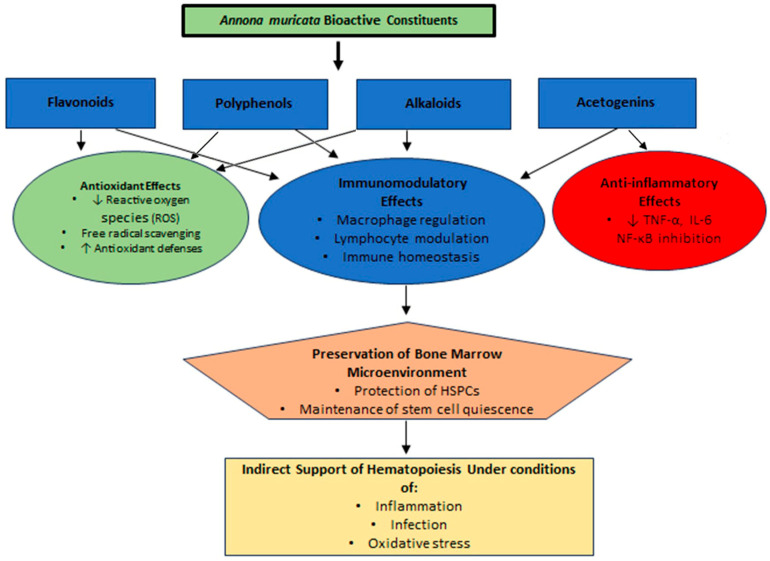
Proposed mechanistic pathways by which *Annona muricata* bioactive components may indirectly influence hematopoietic regulation.

This mechanistic framework provides a rational basis for examining preclinical evidence and supports further investigation into translational applications [20,21,38]. The proposed mechanistic interactions linking *Annona muricata* bioactives to hematopoietic regulation are summarized in Figure 1.

However, these mechanisms remain largely derived from preclinical and indirect evidence, highlighting the need for targeted studies evaluating hematopoietic endpoints.

## 6. Evidence from Preclinical Studies

Preclinical investigations provide the primary scientific foundation for evaluating the potential role of *Annona muricata* in hematopoiesis and blood-related disorders. Although direct studies specifically targeting hematopoietic stem and progenitor cells remain limited, in vitro and in vivo evidence supports indirect hematopoietic relevance through antioxidant, anti-inflammatory, immunomodulatory, and cytoprotective mechanisms. Several experimental studies using *Annona muricata* extracts in cellular and animal models have demonstrated measurable effects on oxidative stress markers, inflammatory pathways, and hematological parameters [82,83,84].

### 6.1. In Vitro Studies

In vitro studies of *Annona muricata* extracts have largely focused on immune cell modulation, oxidative stress attenuation, and cytoprotective effects [21,35]. Polyphenol-rich fractions derived from *A. muricata* leaves have demonstrated significant antioxidant activity in vitro, including reduction in reactive oxygen species and suppression of lipid peroxidation in macrophage, neutrophil, and epithelial cell models [21,35]. These effects are particularly relevant to hematopoiesis, as excessive oxidative stress is known to impair hematopoietic stem cell survival, self-renewal, and lineage commitment [85].

Several experimental studies have reported that *A. muricata* extracts modulate inflammatory signaling pathways, including downregulation of NF-κB activation and reduced production of pro-inflammatory cytokines such as TNF-α and IL-6 [20,21]. Given the established role of these cytokines in suppressing erythropoiesis and leukopoiesis during chronic disease and infection, their inhibition may contribute to a more permissive bone marrow microenvironment. Dose-dependent effects have also been observed in vitro. Low to moderate concentrations of *A. muricata* extracts often exhibit antioxidant and cytoprotective properties, whereas higher concentrations demonstrate cytotoxic effects, particularly against malignant cell lines [38]. This duality underscores the importance of dose optimization when considering hematopoietic relevance, as excessive cytotoxicity could theoretically impair marrow progenitor populations.

### 6.2. Animal Studies

Animal models provide further insight into the hematological effects of *A. muricata* [80,86,87,88]. Several rodent studies have reported changes in hematological parameters following administration of leaf or fruit extracts, likely secondary to modulation of oxidative stress and inflammatory pathways. However, direct assessment of bone marrow structure and hematopoietic stem cell function remains limited in these models. For example, animal studies have reported improvements in hematological indices and reductions in oxidative stress markers following administration of *A. muricata* extracts, supporting a potential role in modulating systemic and marrow-related physiological processes [89].

In experimental models of chemically induced toxicity and inflammation, *A. muricata* extracts have demonstrated protective effects against oxidative tissue damage, preserving cellular integrity in highly proliferative tissues [23]. Although bone marrow histopathology is not consistently evaluated in these studies, stabilization of peripheral blood parameters implies indirect support of marrow function. These findings, while indirect, support a potential link between *A. muricata* bioactivity and hematopoietic regulation, although direct mechanistic studies remain limited. In vitro investigations have further demonstrated modulation of inflammatory mediators and cellular oxidative responses, reinforcing the biological plausibility of these effects [57].

Modulation of leukocyte profiles has also been observed, with normalization of white blood cell differentials reported in inflammatory and infectious models [35]. These effects align with ethnomedicinal claims of enhanced vitality and immune resilience following *A. muricata* consumption and suggest a potential role in immune-related hematopoietic recovery.

While not definitive, these findings support further investigation into potential myeloprotective or adjunctive roles, particularly in oncology settings [90].

### 6.3. Limitations of Preclinical Evidence

Despite encouraging findings, the current preclinical evidence base remains limited in directly assessing hematopoietic outcomes. Most studies were not designed specifically to assess hematopoiesis, and standardized hematological endpoints such as bone marrow cellularity, colony-forming unit assays, or hematopoietic stem cell marker analysis are rarely reported. Additionally, substantial heterogeneity exists with respect to plant part used, extraction method, dosage, and duration of exposure, complicating cross-study comparisons [82,83,84].

Furthermore, the absence of validated human data necessitates cautious interpretation. While preclinical findings support biological plausibility, they do not establish clinical efficacy or safety in hematopoietic disorders. Rigorous translational studies with clearly defined hematological endpoints are required to bridge this gap. Key preclinical findings relevant to hematopoiesis are summarized in Table 1.

## 7. Safety, Toxicity, and Dose Considerations

While *Annona muricata* demonstrates promising biological activity relevant to hematopoiesis, safety considerations remain a critical determinant for its translational potential [91,92]. The therapeutic application of *A. muricata* must be approached with caution, particularly in the context of dose, duration of use, and extract standardization [30,35]. Importantly, much of the available safety data is derived from preclinical models rather than controlled human studies [93,94,95].

### 7.1. Neurotoxicity Concerns

One of the most frequently cited safety concerns associated with *A. muricata* is neurotoxicity linked to annonaceous acetogenins, particularly annonacin [96,97,98]. Epidemiological observations from certain regions have suggested an association between chronic consumption of *A. muricata* products and atypical neurodegenerative syndromes [30,99]. Experimental studies indicate that annonacin impairs mitochondrial complex I activity in neuronal cells, leading to severe energy depletion and neuronal injury at high or prolonged exposure levels [28,29]. In particular, annonacin has been shown to induce tau pathology through microtubule disruption, resulting in somatodendritic accumulation of phosphorylated tau, a hallmark feature observed in atypical Parkinsonian syndromes [28].

However, it is important to distinguish between traditional consumption patterns—often involving diluted infusions or intermittent use and concentrated extracts used in experimental settings. The relevance of neurotoxicity observed at high doses in animal or cellular models to ethnomedicinal use remains uncertain. Nevertheless, these findings underscore the necessity of dose regulation and long-term safety assessment.

### 7.2. Hepatotoxicity and Systemic Toxicity

Animal studies have reported hepatotoxic effects associated with high-dose *A. muricata* extracts, particularly at doses exceeding 300 mg/kg body weight. Observed changes include elevated liver enzymes and histopathological alterations, suggesting potential hepatic stress [35,89,92,100,101]. In contrast, low to moderate doses administered over shorter durations have generally been well-tolerated.

Systemic toxicity appears to be influenced by multiple factors, including extract solvent, plant part used, and duration of administration [94,102,103,104]. Aqueous leaf extracts commonly used in traditional medicine tend to exhibit a more favorable safety profile compared to organic solvent extracts.

### 7.3. Hematological Safety

From a hematopoietic perspective, available preclinical data do not indicate consistent bone marrow suppression at therapeutic doses. In some animal models, *A. muricata* administration has been associated with stabilization or improvement of hematological indices rather than cytopenia [86]. However, the absence of targeted bone marrow toxicity studies limits definitive conclusions.

Given the cytotoxic properties of acetogenins at higher concentrations, the potential for dose-dependent myelotoxicity cannot be excluded. This reinforces the importance of defining safe therapeutic windows before considering clinical application.

### 7.4. Drug–Herb Interactions

Potential interactions between *A. muricata* and conventional medications represent an additional safety concern. Experimental studies suggest that *A. muricata* extracts may influence the pharmacokinetics of certain drugs, including hypoglycemic agents and antihypertensives [99,104,105,106]. In hematological settings, interactions with chemotherapeutic agents or immunomodulators require careful evaluation, particularly in patients with compromised bone marrow function.

### 7.5. Knowledge Gaps and Need for Standardization

A major limitation in assessing the safety of *A. muricata* is the lack of standardized dosing guidelines and extract formulations. Variability in phytochemical composition across geographic regions and preparation methods further complicates risk assessment. Without standardized extracts, reproducibility and safety monitoring remain challenging.

Comprehensive toxicological profiling, including chronic exposure studies and hematopoietic-specific endpoints, is essential before clinical translation. Establishing safe dosage ranges and identifying bioactive compounds responsible for both therapeutic and adverse effects should be prioritized in future research [21,38].

## 8. Translational Implications

The emerging preclinical evidence supporting the biological activity of *Annona muricata* highlights its potential relevance in conditions characterized by impaired hematopoiesis. Although current data remain largely experimental, several translational implications can be considered, particularly in the context of low- and middle-income countries (LMICs), where access to advanced hematological therapies is often limited [91].

### 8.1. Potential Role in Anemia and Bone Marrow Suppression

Anemia remains one of the most prevalent global health challenges, disproportionately affecting populations in LMICs due to nutritional deficiencies, chronic infections, malignancies, and inflammatory disorders [107,108,109,110,111]. In many of these settings, anemia of chronic disease and inflammation-driven marrow suppression are common and poorly addressed by iron supplementation alone [14,68,112].

The antioxidant and anti-inflammatory properties of *A. muricata* suggest a possible supportive role in restoring hematopoietic balance by mitigating oxidative stress and cytokine-mediated suppression within the bone marrow microenvironment. By reducing levels of pro-inflammatory mediators such as TNF-α and IL-6, *A. muricata*-derived compounds may help alleviate inflammation-associated inhibition of erythropoiesis, thereby supporting red blood cell recovery in chronic disease states [35,50,60].

### 8.2. Chemotherapy-Induced Cytopenias and Myelosuppression

Chemotherapy-induced myelosuppression remains a major dose-limiting toxicity in cancer treatment, often leading to treatment delays, increased infection risk, and reduced survival [61,65,66,85]. Current supportive strategies, including growth factor administration and transfusions, are costly and may be inaccessible in resource-limited settings.

In rodent models of carcinogenesis, extracts of *A. muricata* have been shown to reduce pro-inflammatory cytokine levels (e.g., TNF-α, IL-6) and improve antioxidant status, mechanisms relevant to bone marrow microenvironment support [23,47,90,113,114]. Any translational application in oncology must be carefully evaluated to ensure marrow-protective effects do not interfere with antitumor efficacy. Nonetheless, the dual antioxidant and immunomodulatory profile of *A. muricata* warrants further investigation in controlled preclinical models of chemotherapy-associated cytopenias. However, these findings remain preliminary and should be interpreted cautiously pending further validation in controlled models.

### 8.3. Post-Viral and Inflammatory Marrow Dysfunction

Post-viral syndromes are increasingly recognized to involve transient or persistent suppression of bone marrow activity, mediated by immune dysregulation, chronic inflammation, and oxidative stress. Viral infections such as influenza, Ebola, and SARS-CoV-2 have been associated with hematological abnormalities, including anemia, leukopenia, and thrombocytopenia, even after clinical recovery.

The ability of *A. muricata* to modulate inflammatory signaling pathways and oxidative stress suggests potential relevance as a supportive agent in post-viral marrow dysfunction [58,115,116,117,118]. By restoring immune balance and reducing inflammatory inhibition of hematopoietic progenitors, *A. muricata* may contribute to hematological recovery in post-infectious states, particularly where conventional supportive care is limited [21,91].

### 8.4. Relevance to Integrative and Community-Based Care

The widespread cultural acceptance and availability of *A. muricata* across Africa, the Caribbean, and South America make it an attractive candidate for integrative health strategies [17,119]. Translational research that respects ethnomedicinal practices while applying rigorous biomedical evaluation may facilitate the development of standardized, community-acceptable formulations.

Such integrative approaches are particularly relevant in LMICs, where traditional medicine often represents the first line of healthcare. The incorporation of validated plant-based adjuncts into formal health systems has the potential to improve accessibility, adherence, and equity in hematological care, provided safety and efficacy are rigorously established [18,67,119].

### 8.5. Pathway Toward Clinical Translation

For *A. muricata* to progress from experimental evidence to clinical relevance, several translational steps are required. These include the development of standardized extracts with reproducible phytochemical profiles, pharmacokinetic and pharmacodynamic studies, and early-phase clinical trials with clearly defined hematological endpoints.

Potential clinical endpoints may include hemoglobin concentration, reticulocyte counts, white blood cell differentials, platelet indices, and biomarkers of oxidative stress and inflammation. Importantly, such trials should be designed to evaluate *A. muricata* as a supportive or adjunctive agent, rather than as a replacement for established hematological therapies [21,38].

## 9. Challenges and Research Gaps

Despite the promising ethnomedicinal relevance and growing body of preclinical evidence supporting the biological activity of *Annona muricata*, several critical challenges and knowledge gaps must be addressed before its role in hematopoiesis can be reliably defined and translated into clinical practice.

### 9.1. Limited Human Clinical Evidence

A major limitation in the current literature is the absence of well-designed human clinical trials specifically evaluating the hematopoietic effects of *A. muricata* [120]. Most available evidence is derived from in vitro studies, animal models, or indirect pharmacological investigations. While these studies provide important mechanistic insights, they cannot be directly extrapolated to human hematological outcomes.

Clinical endpoints such as hemoglobin recovery, bone marrow cellularity, reticulocyte response, leukocyte reconstitution, and platelet dynamics have not been systematically assessed in human populations exposed to *A. muricata*. This lack of direct clinical evidence remains the most significant barrier to translation.

### 9.2. Variability in Plant Preparation and Dosage

Considerable heterogeneity exists in the preparation of *A. muricata* across studies and traditional practices. Variations include differences in plant parts used (leaves, fruit, bark, seeds), extraction methods (aqueous, ethanolic, methanolic), dosing regimens, and duration of exposure.

Traditional preparations often involve low-dose, intermittent aqueous decoctions, whereas experimental studies frequently employ concentrated extracts with markedly different phytochemical profiles and bioavailability. This variability complicates cross-study comparisons, limits reproducibility, and obscures dose–response relationships relevant to hematopoiesis.

### 9.3. Safety and Toxicity Concerns

Safety considerations, particularly neurotoxicity associated with annonaceous acetogenins such as ‘annonacin,’ represent a critical challenge. Epidemiological observations and experimental studies have linked prolonged or high-dose exposure to mitochondrial dysfunction and neuronal injury [29,30,99]. While traditional use typically involves lower doses and intermittent consumption, the risk associated with standardized or concentrated formulations remains incompletely defined.

From a hematological perspective, the potential for dose-dependent myelotoxicity has not been adequately explored. The absence of long-term toxicity studies incorporating bone marrow-specific endpoints necessitates cautious interpretation of current findings and underscores the need for comprehensive safety profiling.

### 9.4. Methodological Gaps in Hematopoietic Assessment

Many studies investigating *A. muricata* focus primarily on antioxidant, anti-inflammatory, or anticancer outcomes without directly assessing hematopoietic parameters. Critical endpoints such as hematopoietic stem cell integrity, lineage-specific differentiation, marrow niche signaling, and colony-forming capacity are rarely evaluated.

This methodological gap limits the ability to determine whether observed systemic effects translate into meaningful modulation of blood cell production. Future studies must incorporate standardized hematological and bone marrow-specific assays to establish mechanistic relevance.

### 9.5. Regulatory and Ethical Challenges

The integration of ethnomedicinal products into formal healthcare systems faces regulatory hurdles, including inconsistent classification of herbal products, lack of standardized quality control measures, and limited regulatory frameworks for traditional medicines in many regions [4,121].

Ethical considerations surrounding intellectual property rights, benefit-sharing, and protection of indigenous knowledge further complicate translational research. Ensuring equitable collaboration with traditional practitioners and communities is essential to avoid exploitation and preserve the cultural context from which ethnomedicinal knowledge originates.

### 9.6. Need for Interdisciplinary and Translational Research Models

Progress in understanding the hematopoietic relevance of *A. muricata* will require interdisciplinary collaboration bridging ethnomedicine, phytochemistry, hematology, pharmacology, and clinical research. Current siloed approaches limit comprehensive evaluation and slow translation into clinically meaningful applications.

Integrated research models that combine traditional knowledge with rigorous biomedical methodology are necessary to advance *A. muricata* from ethnomedicinal use to evidence-based supportive therapy.

## 10. Conclusions

*Annona muricata* represents a biologically active ethnomedicinal plant with emerging relevance to hematopoiesis and blood-related disorders. Traditional use across Africa, the Caribbean, and South America indicates a longstanding role in managing conditions associated with “blood weakness,” fatigue, immunocompromised state, and post-illness recovery. These ethnomedicinal observations align with contemporary biomedical evidence demonstrating antioxidant, anti-inflammatory, immunomodulatory, and cytoprotective properties of *A. muricata* extracts [20,38].

Preclinical studies suggest that bioactive constituents of *A. muricata*, including flavonoids, polyphenols, and acetogenins, may influence hematopoietic processes indirectly by preserving the bone marrow microenvironment, reducing oxidative stress, and modulating inflammatory cytokine signaling [58,59,61]. Such mechanisms are increasingly recognized as central to the regulation of hematopoietic stem cell function and blood cell production in conditions such as anemia of chronic disease, chemotherapy-induced cytopenias, and post-viral marrow suppression [50,60].

However, despite these mechanistic insights, the current evidence base remains insufficient to support clinical application. The absence of standardized preparations, defined dosing regimens, and controlled human studies limits the translation of *A. muricata* from traditional use to evidence-based hematological practice. Moreover, safety concerns—particularly neurotoxicity associated with annonaceous acetogenins under conditions of high or prolonged exposure—necessitates rigorous toxicological evaluation prior to clinical consideration [28,29,30].

In conclusion, *Annona muricata* should not be regarded as a therapeutic solution, but rather as a scientifically plausible candidate for further investigation. With systematic research, standardized methodologies, and ethical integration of traditional knowledge, *A. muricata* holds potential to contribute meaningfully to future strategies aimed at supporting hematopoiesis and immune recovery, particularly in low-resource and culturally diverse healthcare settings [18,37]. At present, no direct clinical evidence confirms a primary hematopoietic effect.

## 11. Future Directions

Future research on *Annona muricata* should prioritize systematic translation from ethnomedicinal observations and preclinical findings to clinically meaningful hematological outcomes. While existing studies provide biological plausibility, advancing this field will require coordinated efforts that integrate phytochemistry, hematology, pharmacology, and clinical research [91,92].

First, standardization of plant extracts is essential. Future studies should employ well-characterized preparations with defined phytochemical profiles, including quantification of key acetogenins, flavonoids, and polyphenols. Such standardization will enhance reproducibility, allow for meaningful dose–response analyses, and improve cross-study comparability in relation to hematopoietic outcomes [92].

Second, preclinical studies explicitly designed to evaluate hematopoiesis are needed. These investigations should incorporate validated hematological endpoints, including bone marrow cellularity, hematopoietic stem and progenitor cell markers, colony-forming unit assays, and lineage-specific differentiation analyses. This approach would move beyond indirect peripheral blood indices and provide mechanistic clarity at the bone marrow level [58].

Third, early-phase human studies should be cautiously pursued to assess safety, tolerability, and preliminary efficacy. Pilot and feasibility trials may evaluate outcomes such as hemoglobin concentration, reticulocyte counts, leukocyte differentials, platelet indices, and inflammatory biomarkers. Initial clinical exploration may be most appropriate in conditions with high unmet need, including anemia of chronic disease, post-infectious cytopenias, and supportive care during chemotherapy [120].

Fourth, pharmacokinetic and pharmacodynamic studies are required to define bioavailability, metabolism, tissue distribution, and elimination of *A. muricata* bioactive compounds. These data are critical for identifying safe therapeutic windows and minimizing risks associated with cumulative toxicity, particularly neurotoxicity linked to annonaceous acetogenins [99].

Equally important is the ethical integration of ethnomedicinal knowledge into biomedical research frameworks. Collaborative models involving traditional practitioners, scientists, clinicians, and regulatory authorities can help preserve indigenous knowledge, ensure equitable benefit-sharing, and support culturally acceptable interventions [58,107,119].

Finally, multidisciplinary and international collaborations will be key to advancing this field. Partnerships between institutions in low- and middle-income countries and well-resourced research centers can facilitate capacity building, regulatory alignment, and the development of affordable, evidence-based interventions targeting hematological disorders.

Collectively, these future directions provide a structured roadmap for responsibly evaluating *Annona muricata* as a potential supportive agent in hematopoiesis, while maintaining scientific rigor, patient safety, and respect for traditional medical knowledge.

## Figures and Tables

**Table 1:** Summary of reported biological effects of Annona muricata relevant to hematopoiesis.

Bioactive Component/Extract	Reported Biological Effect	Experimental Model	Relevance to Hematopoiesis	References
Leaf polyphenols (quercetin, kaempferol)	Antioxidant activity; reduction in reactive oxygen species; inhibition of lipid peroxidation	In vitro (macrophages, epithelial cells)	Protects hematopoietic stem and progenitor cells (HSPCs) from oxidative stress and preserves bone marrow niche integrity	[21,35]
Crude leaf extract	Anti-inflammatory effects; reduced TNF-α and IL-6; inhibition of NF-κB signaling	In vitro/rodent models	Reduces inflammation-induced suppression of erythropoiesis and leukopoiesis	[20,21]
Acetogenin-containing fractions (e.g., annonacin)	Mitochondrial Complex I inhibition; cytotoxic and immunomodulatory effects; modulates stress-associated hematopoiesis in a dose-dependent manner	In vitro/in vivo	Dose-dependent effects on proliferating cells and hematopoiesis under stress conditions	[28,30]
Whole plant/leaf extracts	Cytoprotective and antioxidant effects	Preclinical studies (animal models)	Indirect support of bone marrow integrity and recovery following oxidative or inflammatory stress	[35,86]

## Data Availability

No new data were created or analysed in this study. All information discussed is sourced from the publications cited in the reference list.
